# Genetic Relatedness of Dolphin Rhabdovirus with Fish Rhabdoviruses

**DOI:** 10.3201/eid2006.131880

**Published:** 2014-06

**Authors:** Jurre Y. Siegers, Marco W.G. van de Bildt, Cornelis E. van Elk, Anita C. Schürch, Noël Tordo, Thijs Kuiken, Rogier Bodewes, Albert D.M.E. Osterhaus

**Affiliations:** Erasmus Medical Centre, Rotterdam, the Netherlands (J.Y. Siegers, M.W.G van de Bildt, C.E. van Elk, A.C. Schürch, T. Kuiken, R. Bodewes, A.D.M.E. Osterhaus);; Dolphinarium Harderwijk, Harderwijk, the Netherlands (C.E. van Elk);; SOS Dolphin Foundation, Harderwijk, the Netherlands (C.E. van Elk);; Institut Pasteur, Paris, France (N. Tordo);; Viroclinics Biosciences, Rotterdam, the Netherlands (A.D.M.E. Osterhaus)

**Keywords:** rhabdovirus, viruses, dolphin, fish, marine mammal, genetic relatedness, genome organization, phylogenetic analysis

**To the Editor:** Rhabdoviruses are enveloped, single-stranded, negative-sense RNA viruses that comprise a large and diverse family in the order *Mononegavirales* and infect arthropods, plants, fish, and mammals. There are 9 genera of rhabdoviruses: *Cytorhabdovirus*, *Ephemerovirus*, *Lyssavirus*, *Novirhabdovirus*, *Nucleorhabdovirus*, *Perhabdovirus*, *Sigmavirus*, *Tibrovirus*, and *Vesiculovirus*. In addition, a substantial number of plant, vertebrate, and invertebrate rhabdoviruses have not been classified (*1*). Three genera (*Novirhabdovirus*, *Perhabdovirus*, and *Vesiculovirus*) comprise members that infect fresh water and marine fish (*2*). Fish rhabdoviruses pose a serious problem for aquaculture because of worldwide outbreaks of disease caused by novirhabdoviruses, perhabdoviruses, and vesiculoviruses (*3*,*4*).

In 1992, a rhabdovirus-like virus was isolated from lung and kidney of a white-beaked dolphin (*Lagenorhynchus albirostris*) that had beached along the coast of the Netherlands (*5*). Although no macroscopic or microscopic lesions were observed at necropsy, negative contrast electron microscopy showed typical rhabdovirus-like, bullet-shaped particles in Vero cell cultures that showed a focal cytopathic effect (*5*). After this rhabdovirus-like virus was injected intracerebrally into brains of 1-day-old suckling mice, they died within 5 days (*5*). We report genetic and phylogenetic characterization of a dolphin rhabdovirus (DRV) and evaluated the seroprevalence of DRV-neutralizing antibodies by using serum samples from various marine mammals collected during a 10-year period (2003–2012).

To characterize DRV, we performed random sequence amplification and deep sequencing with the 454 GS Junior Instrument (Roche, Basel, Switzerland) with DRV-infected Vero cell supernatants as described (*6*). From this analysis, we determined the complete coding sequence of DRV covered by 42,080 of 49,292 reads (minimum coverage 4 reads, average coverage 872 reads).

Genomic termini of DRV were determined by using a 3′ and 5′ rapid amplification of cDNA ends PCR and Sanger sequencing of obtained PCR amplicons. The complete genome of DRV (GenBank accession no. KF958252) consists of 11,141 nt and has a typical rhabdovirus gene arrangement of 5 major open reading frames (ORFs) in the order 3′-nucleoprotein (N), phosphoprotein (P), matrix (M) protein, glycoprotein (G), and large (L) protein-5′ (Figure, panel A, Appendix). No additional ORFs ≥300 nt were detected. Between the major ORFs of DRV, intergenic sequences were present that ranged in size from 34 (P–M) to 83 (G–L) nucleotides. Putative transcription initiation and transcription termination polyadenylation sequences were AACA(G/U) and AUGA_7_, respectively.

**Figure:**
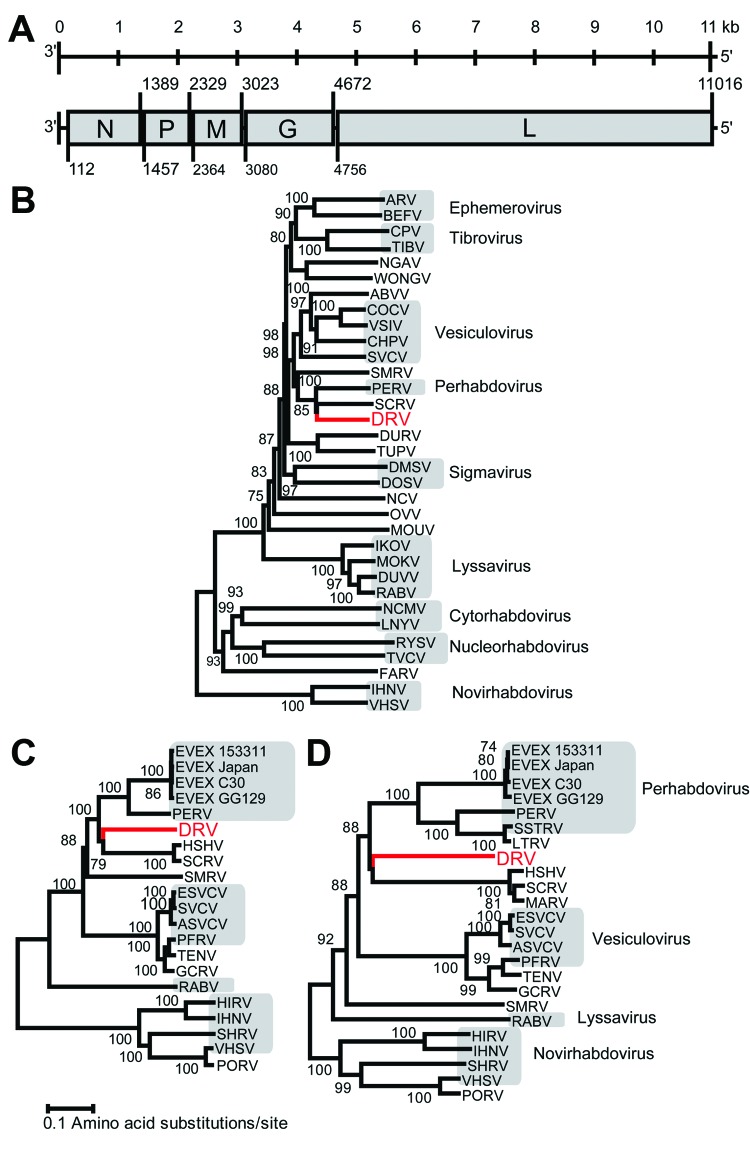
Genome organization and phylogenetic analysis of dolphin rhabovirus (DRV). A) Genome organization of DRV showing locations of major open reading frames and start and stop codons at the nucleotide level. N, nucleoprotein; P, phosphoprotein; M, Matrix; G, glycoprotein; L, large. B) Phylogenetic neighbor-joining tree with P distances (fraction of positions in which 2 sequences differ) and 1,000 bootstrap replicates of the deduced amino acid sequence of complete DRV L gene protein and members of several *Rhabdovirus* genera. C) Neighbor-joining tree with P distances and 1,000 bootstrap replicates of deduced amino acid sequence of complete DRV L gene protein and those of fish rhabdoviruses. D) Neighbor-joining tree with P distances and 1,000 bootstrap replicates of deduced amino acid sequence of complete DRV G gene protein and those of fish rhabdoviruses. DRV is indicated in red and bootstrap support values ≥70 are shown. Lengths of branches are drawn to a scale of amino acid substitutions per site, alignment of several genera was based on a 746-aa segment, and alignment of fish rhabdoviruses was based on a 1,285-aa segment for the L gene and a 227-aa fragment for the G gene. Gray shading indicates associated genus. Nucleotide GenBank accession numbers (in parentheses) used to generate phylogenetic trees are as follows: ARV, Adelaide River virus (JN935380); BEFV, bovine ephemeral fever virus (NC_002526); CPV, coastal plains virus (GQ294473); TIBV, Tibrogargan virus (GQ294472); NGAV, Ngaingan virus (NC_013955); WONGV, Wongabel virus (NC_011639); ABVV, American bat vesiculovirus (NC_022755); COCV, Cocal virus Indiana 2 virus (EU373657); VSIV, vesicular stomatitis virus Indiana (NC_001560); CHPV, Chandipura virus (HM627187); SVCV, spring viremia of carp virus (NC_002803); SMRV, *Scophthalmus maximus* rhabdovirus (HQ003891); PERV, perch rhabdovirus (NC_020803); SCRV, *Siniperca chuatsi* rhabdovirus (NC_008514); DURV, Durham virus (FJ952155); TUPV, Tupaia virus (NC_007020); DMSV, *Drosophila melanogaster* sigma virus (AM689309); DOSV, *Drosophila obscura* sigma virus (GQ410979); NCV, North Creek virus (KF360973); OVV, Oak-Vale virus (JF705876); MOUV, Moussa virus (FJ985749); IKOV, Ikoma lyssavirus (JX193798); MOKV, Mokola virus (NC_006429); DUVV, Duvenhage virus (JN986749); RABV, rabies virus (AF499686); NCMV, northern cereal mosaic virus (NC_002251); LNYV, lettuce necrotic yellows virus (NC_007642); RYSV, rice yellow stunt virus (NC_003746); TVCV, Taro vein chlorosisvirus (NC_006942); FARV, Farmington virus (KC602379); IHNV, infectious hematopoietic necrosis virus (L40883); VHSV, viral hemorrhagic septicemia virus (AB672614); EVEX 153311, eel virus European X 153311 (FN557213); EVEX Japan, eel virus European X Japan (JX827265); EVEX C30, eel virus European X C30 virus (JN639009); EVEX GG129, eel virus European X GG129 (JN639010); HSHV, hybrid snakehead virus (KC519324); ESVCV, European spring viremia of carp virus (AJ318079); ASVCV, Asian spring viremia of carp virus (DQ097384); PFRV, pike fry rhabdovirus (FJ872827); TENV, Tench rhabdovirus (KC113517); GCRV, grass carp rhabdovirus (KC113518); HIRV, Hirame rhabdovirus (NC_005093); SHRV, snakehead rhabdovirus (NC_000903); PORV, *Paralichthys olivaceus* rhabdovirus (KC685626); SSTRV, Swedish sea trout rhabdovirus (AAL38523); LTRV, lake trout rhabdovirus (AF434991); MARV, *Monopterus albus* rhabdovirus (AGZ15720).

The deduced amino acid sequence of genes of DRV and several other rhabdoviruses were aligned by using MUSCLE in MEGA5 version 5.2) (*7*). Ambiguous aligned regions were removed by using the Gblocks program (*8*). Phylogenetic analysis of the L and G genes was performed by using the neighbor-joining method in MEGA5 (*7*). This analysis showed that DRV is most closely related to fish rhabdoviruses of the genera *Perhabdovirus* and *Vesiculovirus* and unassigned fish rhabdoviruses with strong bootstrap support (Figure, panels B–D, Appendix).

Deduced amino acid sequences of the 5 major genes had the highest, although weak, homology with those of various fish rhabdoviruses by pairwise identity analyses: N (48%) with hybrid snakehead virus (HSHV), *Monopterus albus* rhabdovirus (MARV), and *Siniperca chautsi rhabdovirus* (SCRV); P (18%–20%) with eel virus European X (EVEX), HSHV, MARV, and SCRV; M (27%–33%) with lake trout rhabdovirus, Swedish sea trout rhabdovirus, and EVEX; G (30%–32%) with perch rhabdovirus, lake trout rhabdovirus, Swedish sea trout rhabdovirus, HSHV, MARV, SCRV, and EVEX; and L (54%–56%) with perch rhabdovirus, HSHV, and EVEX. This close relationship with fish rhabdoviruses is surprising because DRV was isolated from tissues of a mammal and propagated in mammalian cell lines at 37°C, which does not occur with related viruses isolated from fish.

To evaluate whether DRV or related viruses circulate among species of cetaceans, we performed serologic screening by using a virus neutralization assay as described (*5*). The specificity of this assay was tested by using a panel of rhabdovirus-specific antisera obtained from cetaceans of various species (*5*). The serum samples had been collected for diagnostic purposes from mainly juvenile cetaceans stranded along the coast of the Netherlands during 2003–2012. These species included 2 Atlantic white-sided dolphins (*Lagenorhynchus acutus*)*,* 79 harbor porpoises (*Phocoena phocoena*)*,* 9 striped dolphins (*Stenella coeruleoabla*), and 6 white-beaked dolphins (*Lagenorhynchus aIbirostris*). Serum samples from 145 bottlenose dolphins (*Tursiops truncates*) from the collection of the Dolphinarium Harderwijk (Harderwijk, the Netherlands) were also tested. DRV-neutralizing antibodies were detected in serum samples from 1 bottlenose dolphin (7%), 5 striped dolphins (55%), 1 white-beaked dolphin (17%), and 3 harbor porpoises (4%). These results suggested that DRV or closely related viruses continue to infect members of cetacean species (*6*).

Although rhabdovirus evolutionary pathways are complicated (*9*), our analysis suggests that DRV is a possible derivative of fish rhabdoviruses. DRV might have originated from an unidentified fish rhabdovirus and might cycle between fish and marine mammals, similar to that suggested for cycling of vesicular stomatitis virus between arthropods and terrestrial mammals (*10*). Future analyses of sequences from other marine mammal rhabdovirus sequences might support the validity of our phylogenetic analysis and result in creation of a new group containing marine mammal rhabdoviruses.
